# Self-Assembly Nanostructure of Myristoylated ω-Conotoxin MVIIA Increases the Duration of Efficacy and Reduces Side Effects

**DOI:** 10.3390/md21040229

**Published:** 2023-04-01

**Authors:** Xiufang Ding, Yue Wang, Sida Zhang, Ruihua Zhang, Dong Chen, Long Chen, Yu Zhang, Shi-Zhong Luo, Jianfu Xu, Chengxin Pei

**Affiliations:** 1 State key Laboratory of NBC Protection for Civilian, Beijing 102205, China; 13552909516@163.com (X.D.); wy1603762885@163.com (Y.W.); stars5123@163.com (S.Z.); ch_ever@126.com (D.C.); zyuyuyu168@163.com (Y.Z.); 2 Beijing Key Laboratory of Bioprocess, College of Life Science and Technology, Beijing University of Chemical Technology, Beijing 100029, China; chenlong@mail.buct.edu.cn (L.C.); luosz@mail.buct.edu.cn (S.-Z.L.)

**Keywords:** self-assembly, conotoxin, analgesia, myristoylation, micelles

## Abstract

Chronic pain is one of the most prevalent health problems worldwide. An alternative to suppress or alleviate chronic pain is the use of peptide drugs that block N-type Ca^2+^ channels (Ca_v_2.2), such as ω-conotoxin MVIIA. Nevertheless, the narrow therapeutic window, severe neurological side effects and low stability associated with peptide MVIIA have restricted its widespread use. Fortunately, self-assembly endows the peptide with high stability and multiple functions, which can effectively control its release to prolong its duration of action. Inspired by this, MVIIA was modified with appropriate fatty acid chains to render it amphiphilic and easier to self-assemble. In this paper, an N-terminal myristoylated MVIIA (Myr-MVIIA, medium carbon chain length) was designed and prepared to undergo self-assembly. The present results indicated that Myr-MVIIA can self-assemble into micelles. Self-assembled micelles formed by Myr-MVIIA at higher concentrations than MVIIA can prolong the duration of the analgesic effect and significantly reduce or even eliminate the side effects of tremor and coordinated motor dysfunction in mice.

## 1. Introduction

Chronic pain is an intermittent or persistent pain sensation that persists for more than three months [1]. It poses a serious threat to over 30% of the global population and imposes an enormous personal, social and economic burden [2]. In spite of the range of painkillers available, the chronic condition remains intractable, and it is rare for symptoms to disappear completely [3,4].

Drugs that target voltage-gated calcium channels (VGCCs) to treat chronic pain are a focal point in global drug discovery [3,4,5,6,7,8]. VGCCs have been implicated in important physiological processes, including neurotransmitter release and pain signaling. The N-type VGCC (Ca_v_2.2 channel) is the central pain target among the different types of VGCCs [9]. The primary pharmacological family for targeting the Ca_v_2.2 channel are the ω-conotoxins. Blockade of the Ca_v_2.2 channel by ω-conotoxin prevents the perception of painful stimuli by inhibiting the transmission of neurotransmitters (such as substance P and glutamate) [9,10]. The best-known of the many calcium-channel-blocking ω-conotoxins is ω-conotoxin MVIIA, also called ziconotide (Prialt®). Highly effective and non-addictive, MVIIA was the first peptide drug to receive approval from the US Food and Drug Administration (FDA) for the management of refractory pain [11].

Although MVIIA has been shown to be a potent analgesic, there are also some limitations to its use. These include poor bioavailability and stability [12], a narrow therapeutic window [13] and severe unexpected side effects, even at doses used for analgesic treatment [14,15]. It is therefore possible to modify the structure of the drug to improve its stability and reduce its side effects.

In recent years, there has been a great deal of interest in lipidized peptide drugs to improve their bioavailability, increase their stability and prolong their half-life [16,17,18]. The lipidization of a hydrophilic peptide drug gives the molecule an amphiphilic characteristic, which enhances its tendency to self-assemble [19]. The self-assembled nanostructure of amphiphilic peptides can enhance the bioavailability and/or stability, and limit the side effects of drugs [20,21]. For example, insulin detemir containing myristic acid side chains was reversibly bound to human serum albumin and promoted the self-assembly of insulin, which slowed the diffusion of insulin in vivo and prolonged the duration of action of the drug [22,23,24]. In addition, neither extremely short (C8 and below) nor long (C18 and above) fatty acid chains could induce peptide self-assembly, i.e., fatty acid chains of an appropriate length were important for the assembly of peptides into stable and ordered structures [25]. Therefore, an N-terminal myristoylated MVIIA (Myr-MVIIA, moderate carbon chain length) was designed with the expectation that its self-assembled nanostructure would improve the drug stability and reduce the side effects by slowing the drug release.

## 2. Results

### 2.1. Myristoylated MVIIA Self-Assembled into Supramolecular Nanostructures

**Figure 1:**
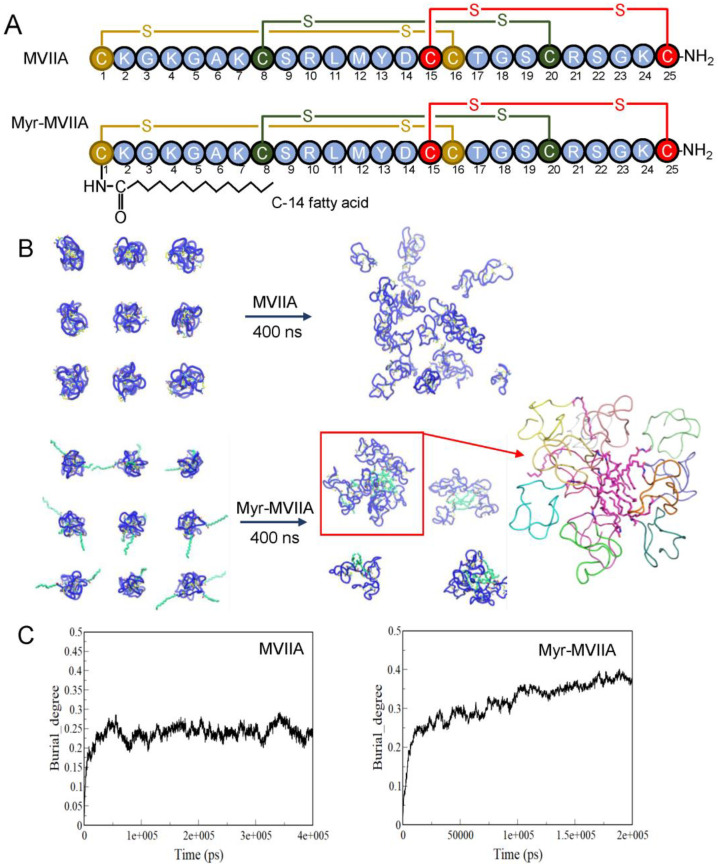
Design and self-assembly simulation of N-terminal myristoylated MVIIA (Myr-MVIIA). (**A**) Structures of MVIIA and Myr-MVIIA with disulfide bonds. (**B**) Simulation snapshots of the assembly of MVIIA and Myr-MVIIA in the initial (0 ns) and final states (400 ns). (**C**) Variation of the degree of burial with time during MVIIA and Myr-MVIIA simulations.

The peptide drug MVIIA contains 25 residues, of which 6 cysteine residues form 3 pairs of disulfide bonds between them [26]. The sequence of MVIIA is shown in Figure 1A. MVIIA is a highly water-soluble peptide. To render MVIIA more hydrophobic to facilitate its nanoassembly in aqueous solution, myristic acid, a 14-carbon fatty acid, was attached to its N-terminus via an amide bond (Figure 1A). Molecular dynamics (MD) simulations were used to show the effect of the myristic acid modification on the self-assembly of the peptide MVIIA. Two parallel simulation systems were set up, as shown in Figure 1B. After 400 ns of simulation, Myr-MVIIA formed distinct aggregates compared to the dispersed peptide MVIIA. The burial degree of Myr-MVIIA was also higher than that of MVIIA, indicating that the degree of aggregation of Myr-MVIIA was higher than that of MVIIA. When Myr-MVIIA was simulated for only 200 ns, the degree of burial continued to increase (Figure 1C). All this suggested that Myr-MVIIA aggregated more easily; thus, the N-terminal myristic acid modification of MVIIA theoretically promotes self-assembly. 

**Figure 2:**
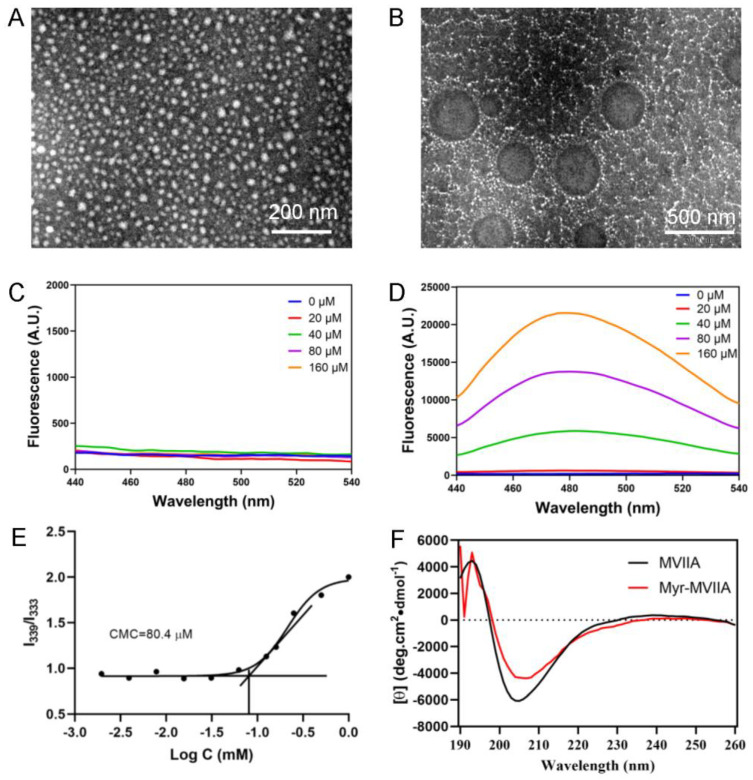
Characterization of aggregates formed by MVIIA and Myr-MVIIA segments. Transmission electron microscopy (TEM) images of MVIIA (**A**) and Myr-MVIIA (**B**) aggregates at 37 °C for 24 h. The anionic 1-anilinonaphthalene-8-sulfonic acid (ANS) fluorescence traces for MVIIA (**C**) and Myr-MVIIA (**D**). (**E**) The critical micelle concentration (CMC) value of Myr-MVIIA determined by pyrene fluorescence spectra. (**F**) Circular dichroism spectra of MVIIA and Myr-MVIIA.

Subsequently, the peptides MVIIA and Myr-MVIIA were incubated for 24 h in the same self-assembly environment, i.e., a physiological pH (pH = 7.4) and temperature (37 °C). The peptide samples were negatively stained, and the morphology of the peptides’ self-assembly was characterized using transmission electron microscopy (TEM, Figure 2A,B). It was shown that only Myr-MVIIA can form micelles with diameters of 170–372 nm (Figure 2B). Instead, the peptide MVIIA formed spherical oligomers with diameters of 14–40 nm under the same conditions (Figure 2A). The particle size of the peptides was further examined by the dynamic light scattering method. The particle size of MVIIA was 48.74 ± 16.07 nm and that of Myr-MVIIA was 301.83 ± 98.09 nm (Table S1). This was in general agreement with the results observed for the dried samples investigated using TEM. Anionic 1-anilinonaphthalene-8-sulfonic acid (ANS), which probes the hydrophobicity of molecules, is a well-known molecular probe that detects the ability of peptides or proteins to aggregate. As shown in Figure 2D, there was a significant increase in the fluorescence intensity with the increasing peptide Myr-MVIIA concentration. In addition, the highest peak of ANS emissions shifted from 510 nm to near 480 nm, i.e., a blue shift in its own emission spectrum, demonstrating that ANS bound to the aggregated Myr-MVIIA. Figure 2C,D further shows that the self-assembly ability of Myr-MVIIA was higher than that of MVIIA. As determined by pyrene fluorescence, the critical micelle concentration (CMC) of the peptide Myr-MVIIA was 80.4 µM (Figure 2E). Circular dichroism (CD) was employed to determine the secondary structure of the peptides. As shown in Figure 2F, both MVIIA and Myr-MVIIA were found to adopt beta-sheet structures, as characterized by a maximum near 195 nm and a minimum near 205 nm.

### 2.2. Inhibition of Ca_v_2.2 Channel Currents Induced by MVIIA and Myr-MVIIA

**Figure 3:**
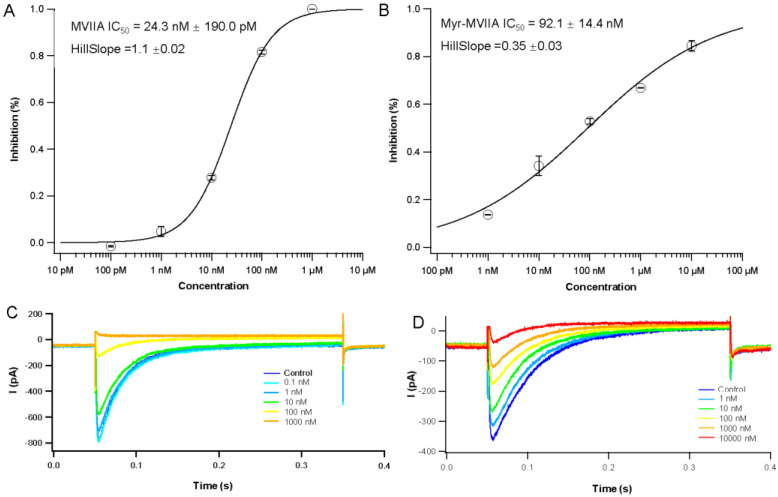
Electrophysiological properties of MVIIA and Myr-MVIIA. The concentration–response curves for MVIIA (**A**) and Myr-MVIIA (**B**). The IC_50_ and Hill slope values for MVIIA and Myr-MVIIA are presented in the graph. Data are presented as mean ± SEM, *n* = 3. Representative traces of N-type Ca^2+^ currents (control) blocked by MVIIA (**C**) and Myr-MVIIA (**D**) in Chinese hamster ovary (CHO) cells.

The ω-conotoxin MVIIA is a well-known selective and potent antagonist of the Ca_v_2.2 channel [27]. In this paper, the peak Ca^2+^ currents (ICa) of stably overexpressed Ca_v_2.2 channels in Chinese hamster ovary (CHO) cells were recorded to observe the effects of drugs on the Ca_v_2.2 currents. A total of 1 µM MVIIA and Myr-MVIIA reduced the ICa amplitude by 100.00 ± 0.00% and 66.83 ± 0.16%, respectively (*n* = 3) (Figure 3A,B). The peptide Myr-MVIIA inhibited the Ca_v_2.2 channel currents less than MVIIA (Figure 3A–D). The IC_50_ of the concentration–response curve for MVIIA’s inhibition of Ca_v_2.2 was 24.3 nM ± 190.0 pM (Figure 3A), whereas the IC_50_ of Myr-MVIIA was 92.1 nM ± 14.4 nM (Figure 3B). Thus, Myr-MVIIA had a higher IC_50_ than MVIIA, suggesting that the addition of the myristic acid chain may weaken its binding ability to Ca_v_2.2.

### 2.3. Analgesic Effects of Peptides on Acetic-Acid-Induced Visceral Pain in Mice

**Figure 4:**
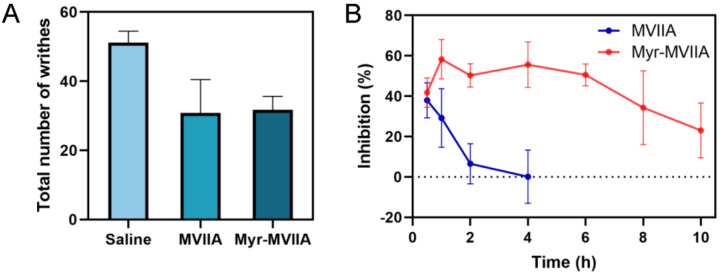
Antinociceptive effect in acetic-acid-induced writhing. (**A**) The number of writhes treated by MVIIA (1 nmol/kg) and Myr-MVIIA (1 nmol/kg). (**B**) Changes in the antinociceptive effect (%) produced by MVIIA (1 nmol/kg) and Myr-MVIIA (16 nmol/kg) at different times after administration. Data are presented as mean ± SEM, *n* = 8.

The acetic-acid-induced writhing test of mice is a commonly-used model to evaluate the analgesic effects of drugs. First, the analgesic effects of equal molar concentrations of the peptides MVIIA and Myr-MVIIA administered as one dose (1 nmol/kg) were tested. After the intracerebroventricular (ICV) injection of the peptides into the mice for 30 min, an intraperitoneal injection of acetic acid was performed to count the number of writhing movements in the mice. As shown in Figure 4A, the number of writhing movements for the peptides MVIIA and Myr-MVIIA were very close to each other, demonstrating that both peptides had similar analgesic effects at a dose of 1 nmol/kg. The peptide concentration at the injected dose of 1 nmol/kg was 20 µM, and the critical micelle concentration of Myr-MVIIA was 80.4 µM (Figure 2E). Therefore, the peptide Myr-MVIIA did not aggregate into the micelles at this concentration. Since the self-assemblies tended to be highly stable and slow-releasing, the duration of the analgesic effect was further investigated with a higher concentration (320 µM) of the assembled Myr-MVIIA (a dose of 16 nmol/kg). However, MVIIA could not be selected at too high a concentration because, during the experiment, the tremors in the mice at high concentrations of MVIIA were very severe and this interfered with the assessment of its analgesic effect. For both peptides, durations of 1, 2, 4, 6, 8 and 10 h were chosen before acetic acid pain stimulation. As shown in Figure 4B, Myr-MVIIA (16 nmol/kg) exhibited the highest inhibition rate of writhing (58.33% ± 9.77%) at 1 h post-administration and then showed a gradual decrease for up to 10 h post-administration. In contrast, MVIIA (1 nmol/kg) showed the highest inhibition rate of writhing (37.96% ± 8.67%) at 30 min after administration but dramatically decreased to 0.15% ± 13.17% at 4 h. Myr-MVIIA maintained a high inhibition rate of writhing for a long duration after administration, suggesting that the self-assembled micelles formed by Myr-MVIIA at higher concentrations than MVIIA may prolong the duration of the analgesic effect.

### 2.4. Coordinated Locomotion Effects of Peptides

**Figure 5:**
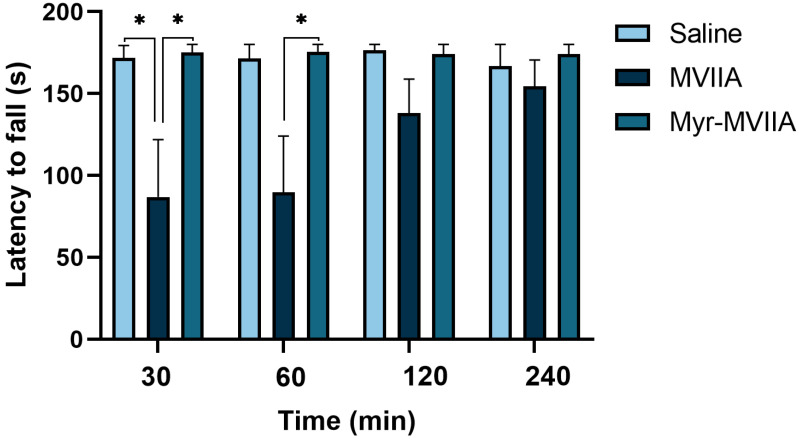
Effects of MVIIA and Myr-MVIIA on coordinated locomotion. After MVIIA (1 nmol/kg), Myr-MVIIA (16 nmol/kg) or saline (control) injections for 30, 60, 120 and 240 min, the mice were placed on the rotarod. Data are presented as mean ± SEM, *n* = 6. * *p* ≤ 0.05 by one-way ANOVA with Dunnett’s T3 multiple comparison test.

It has been shown that MVIIA caused motor dysfunction when used as an analgesic, and this side effect became more severe with increasing doses of MVIIA [28,29]. In this study, the rotarod test was performed to evaluate the effects of MVIIA (1 nmol/kg) and Myr-MVIIA (16 nmol/kg) on coordinated locomotor function. After 30 min of injection (ICV), the residence times of the mice on the rotarod were 171.67 ± 7.75, 86.83 ± 35.09 and 175.00 ± 5.00 s for saline, MVIIA and Myr-MVIIA, respectively (Figure 5). After 4 h of injection (ICV), the residence times of the mice on the rotarod were 166.83 ± 13.17, 154.50 ± 16.13 and 180.00 ± 0.00 s for saline, MVIIA and Myr-MVIIA, respectively. The effect of MVIIA on coordinated locomotion in the mice was significant at 30–60 min and gradually decreased from 2 to 4 h. In contrast, Myr-MVIIA did not affect coordinated locomotion in the mice. All these findings indicated that the injection (ICV) of MVIIA interfered with coordinated locomotion in the mice, whereas Myr-MVIIA did not.

### 2.5. Tremor Symptoms in Mice Induced by Peptides

**Figure 6:**
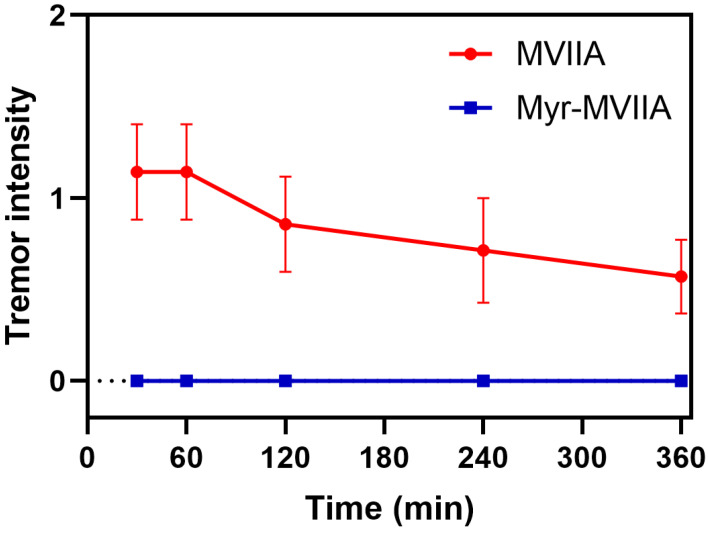
Effects of MVIIA and Myr-MVIIA on tremor in mice. MVIIA (1 nmol/kg) and Myr-MVIIA (16 nmol/kg) were administered to mice for 30, 60, 120, 240 and 360 min, after which the tremor intensity was assessed. Data are presented as mean ± SEM, *n* = 8.

MVIIA is known as a “shaker” peptide, and tremor is a typical side effect of MVIIA [29]. At 30, 60, 120, 240 and 360 min after ICV administration, the intensity of tremors in mice induced by MVIIA/Myr-MVIIA was scored on a 4-point-graded scale, i.e., 0: normal (no tremor); 1: mild (tremors in limited areas of the body, such as the head, neck, forelimbs and tail); 2: moderate (tremors in multiple areas of the body, including the head, upper body and trunk); and 3: severe (severe tremors throughout the whole body) [30]. MVIIA (1 nmol/kg) had the highest tremor intensity at 30–60 min, after which it gradually decreased (Figure 6). However, there were no tremor symptoms in the mice after Myr-MVIIA post-injection (ICV), even at higher doses (16 nmol/kg). Thus, the assembled Myr-MVIIA micelles reduced or even eliminated the side effects of tremor.

### 2.6. Serum Stability of Peptides

**Figure 7:**
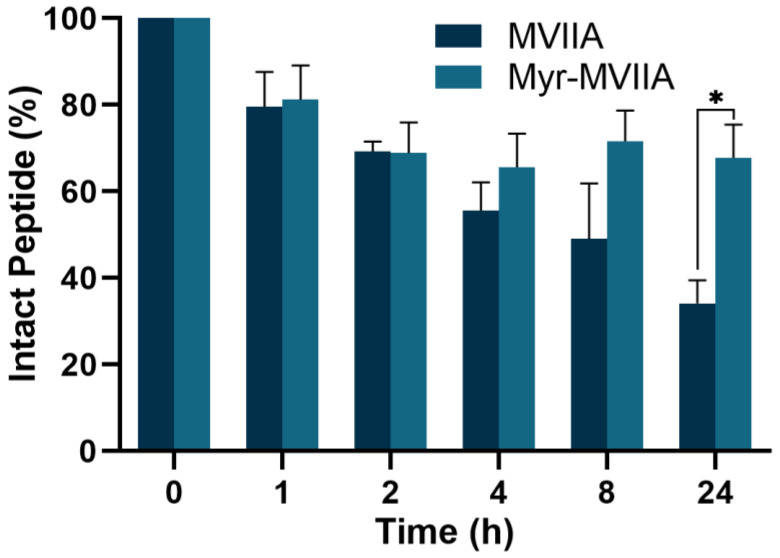
Degradation kinetics of peptides MVIIA and Myr-MVIIA in human serum. Data are presented as mean ± SEM, *n* = 4. * *p* ≤ 0.05 by *t*-test.

The main obstacle to the clinical application of bioactive peptides is the problem of their stability in the organism. There are several reasons for the poor in vivo stability of peptides, including glomerular filtration, degradation by in vivo enzymes, absorption by the reticuloendothelial system, etc. [31,32]. One of the advantages of peptide self-assembly is improved serum stability. As shown in Figure 7, the half-life of the peptide MVIIA in 10% serum was approximately 8 h. However, the self-assembled peptide Myr-MVIIA was more stable in human serum, with 67.80% of the peptide Myr-MVIIA remaining intact after 24 h. This indicated that the self-assembled micelles could protect the peptide from serum protease degradation and that the peptide Myr-MVIIA has high application potential.

## 3. Discussion

MVIIA exerts its analgesic effects by blocking N-type calcium channels. Therefore, we evaluated the effect of the in vitro electrophysiological activity of Myr-MVIIA on N-type calcium channels. Myr-MVIIA retained its inhibitory activity against Ca_v_2.2, although with a slightly reduced potency compared to MVIIA (Figure 3). This suggested that the N-terminal myristic acid chain “tail” may slightly affect the folding structure of MVIIA, which in turn affects the binding of MVIIA amino acids to Ca_v_2.2. Nevertheless, the micelle structure formed by the self-assembly of Myr-MVIIA showed other advantages. This study used intraperitoneal injections of acetic acid to simulate chronic pain stimulation. Equal doses of MVIIA and Myr-MVIIA had similar strengths of analgesic effects in this chronic pain model. The injection (ICV) of high concentrations of Myr-MVIIA (16 nmol/kg) resulted in a 23.08% ± 13.63% inhibition rate of writhing at 10 h. However, MVIIA was used at an appropriate dose (1 nmol/kg) to evaluate its analgesic duration, as an excessively high dose caused severe tremor and coordination dysfunction. After 4 h of MVIIA injection, the inhibition rate of writhing was only 0.15% ± 13.17%, indicating that little analgesic effect remained. In contrast, a high dose of injected Myr-MVIIA prolonged the duration of the analgesic effect and did not cause the side effects of tremor and coordinated motor dysfunction. Self-assembled nanostructures were considered to control the sustained release and stability of peptide drugs [33]. In addition, self-assembled nanostructures reduced the affinity of proteases for peptides and protected them from protease cleavage [34]. Therefore, we speculated that the self-assembly of Myr-MVIIA plays a major role in this process. First, the self-assembly of the peptide Myr-MVIIA enables the slow release of peptide drugs in vivo. Second, the self-assembled micelles reduce the affinity of proteases for Myr-MVIIA. Some amino acid sites that are easily cut by proteases are wrapped in the assembly, making it difficult for the peptides to be enzymatically hydrolyzed.

In conclusion, the designed N-terminal myristoylated MVIIA can self-assemble into micelles. Self-assembled micelles formed by Myr-MVIIA at higher concentrations than MVIIA can prolong the duration of the analgesic effect and significantly reduce or even eliminate the side effects of tremor and coordinated motor dysfunction in mice. In addition, the results of this work establish a basis for the future development of carrier-free drug delivery systems.

## 4. Materials and Methods

### 4.1. Materials and Reagents

The two peptides (MVIIA and Myr-MVIIA) used in this study (>98% purity) were purchased from Taijia Co., Ltd. (Hangzhou, China). A CHO cell line stably overexpressing Ca_v_2.2 channels was obtained from ICE Bioscience Inc. (Beijing, China). All other chemical or biological reagents were obtained from Sigma-Aldrich Co. (St. Louis, MO, USA) unless otherwise indicated.

### 4.2. MD Simulation

The simulations for this study were conducted using GROMACS-2019 [35]. The structure of MVIIA was obtained from the RCSB Protein Data Bank (PDB ID: 10MG), while the structure of Myr-MVIIA was obtained from the Charmm-gui website (https://charmm-gui.org/, accessed on 12 May 2021) [36]. A total of 27 peptide molecules were simulated using the Charmm36m protein force field and the TIP3P water model. The box size for MVIIA was 9 × 9 × 9 nm^3^, while the box size for Myr-MVIIA was 10.5 × 10.5 × 10.5 nm^3^. Energy minimization was performed for 5000 steps using the steep descent method. The Nose–Hoover coupling method was used for NVT equilibration with a reference temperature of 303.15 K and an equilibration time within 200 ps. The Parrinello–Rahman coupling method was adopted for NPT equilibration with a compression value of 4.5 × 10^−5^ bar^−1^, a reference pressure of 1.0 bar and an equilibration time of 200 ps. Unrestricted MD simulations were run for 400 ns. VMD software was used to visualize the protein structure.

### 4.3. Peptide Synthesis and Peptide Sample Preparation

A number of synthetic fatty-acid-modified peptides have been reported, such as myristoylated human prolactin-releasing peptide 20 (PrRP20) and palmitoylated PrRP31 [16], endomorphin-1 (EM) derivatives C18-SS-EM1 and C18-CONH-EM1 [37], myristoylated human alpha-defensin 5 (HD5) [38], N-myristoylated collapsin response mediator protein 2 (CRMP2) [39], etc. MVIIA and Myr-MVIIA were synthesized by the classical Fmoc solid-phase synthesis method. Myr-MVIIA was synthesized by the targeted introduction of myristic acid at the N-terminus during the synthesis process. Briefly, the synthetic peptides were cleaved from the resin after deprotection and coupling with Fmoc-AA-OH using Fmoc-Linker AM resin as a solid-phase substrate. The peptides formed disulfide bonds by oxidative folding. Finally, the peptides were purified by HPLC, and the eluates were collected and lyophilized to obtain the pure peptide powder. The HPLC and ESI-MS of the peptides MVIIA and Myr-MVIIA are shown in Figure S1 and Figure S2, respectively. The peptide purity for MVIIA and Myr-MVIIA was 99.6% and 98.9%, respectively. The molecular mass for MVIIA and Myr-MVIIA was 2639.2 and 2849.5, respectively (Figures S1 and S2).

The peptide MVIIA was dissolved in aqueous media at a concentration of 10 mM as the initial solution, while the peptide Myr-MVIIA was dissolved in 10% *v/v* DMSO/water at the same concentration as the initial solution. All working solutions for TEM, the ANS binding assay, CMC assay and CD spectroscopy were diluted from the initial solution to the indicated concentrations. 

### 4.4. TEM Measurements

Working solutions of the peptides MVIIA and Myr-MVIIA were diluted with water from the initial solution to a concentration of 500 µM. The peptide working solution was incubated at 37 °C for 24 h. A total of 8 µL of the finished incubation solution of MVIIA and Myr-MVIIA was added onto a carbon-coated copper grid by droplet and held for approximately 5 min in each case. Then, 8 µL of 2% aqueous sodium phosphotungstate solutions (pH = 6.5) was dropped onto the copper grid to stain the MVIIA and Myr-MVIIA samples for approximately 1 min. Images were captured on a JEOL JEM1200EX by TEM at 100 kV.

### 4.5. Dynamic Light Scattering 

Working solutions of the peptides MVIIA and Myr-MVIIA were diluted with 10 mM PBS (pH = 7.4) from the initial solution to a concentration of 500 µM. The peptide working solution was incubated at 37 °C for 24 h. The particle size of the peptides was measured on a Malvern Zetasizer Nano™ (London, UK) instrument. Polydispersity index (PDI) values were calculated using the Malvern Zetasizer Nano™ instrument software (v5.10, London, UK).

### 4.6. ANS Binding Assay

The as-prepared peptide MVIIA and Myr-MVIIA working solutions were obtained by diluting the initial peptide solution with 10 mM PBS, respectively, and the final peptide concentrations were 20 µM, 40 µM, 80 µM and 160 µM. After the diluted peptide solution was incubated overnight at 37 °C, ANS was added at a final concentration of 20 µM. The fluorescence spectra of the different concentrations of MVIIA and Myr-MVIIA samples were measured using a Hitachi F-4600 fluorescence spectrophotometer (Tokyo, Japan). The excitation wavelength was selected as 369 nm and the excitation spectrum was in the range of 440–540 nm.

### 4.7. Determination of CMC

The CMC of the peptide Myr-MVIIA was detected using the fluorescent dye pyrene. Working solutions of Myr-MVIIA were prepared by diluting the initial solution of peptide Myr-MVIIA with 10 mM PBS (pH = 7.4) to obtain a final peptide concentration in the range of 1.95–1000 µM. The pyrene was dissolved in acetone (0.035 mg/mL, 20 µL) and carefully dried with N_2_. Then, 1 mL of diluted peptide working solution was added and left overnight to ensure that Myr-MVIIA was sufficiently bound to pyrene to reach equilibrium. The fluorescence spectra of the different concentrations of Myr-MVIIA samples were measured using a Hitachi F-4600 fluorescence spectrophotometer (Tokyo, Japan). The emission wavelength was chosen to be 395 nm and the excitation spectrum was in the range of 300–380 nm with a bandwidth of 5 nm. The CMC values were obtained by plotting I339/I333 versus the peptide concentration.

### 4.8. CD Spectroscopy

CD is commonly employed to detect the secondary structures of peptides. Initial solutions of the peptides MVIIA and Myr-MVIIA were diluted to 80 µM with ultrapure water. After the peptide was incubated at 37 °C for 24 h, CD was performed using a Jasco J-810 spectropolarimeter (Jasco Co., Tokyo, Japan). After obtaining the millidegrees (θ), the molar residual ellipticity (MRE) value was expressed by the formula [θ] = θ × M/(l × c × nr), where M represents the molar mass (g/mol) of MVIIA/Myr-MVIIA, l represents the path length (mm), c represents the concentration (mg/mL) of MVIIA/Myr-MVIIA, and nr represents the number of residues.

### 4.9. Electrophysiology

CHO cells with a stable overexpression of the Ca_v_2.2 channel were used, having been obtained from ICE Bioscience Inc. (Beijing, China). The cells were cultured in 89% (*v/v*) Ham’s F-12 medium (Gibco) and 10% (*v/v*) fetal bovine serum (Gibco), 100 µg/mL Zeocin, 800 µg/mL G418 and 200 µg/mL Hygromycin B at 37 °C in 5% CO_2_. Prior to patch-clamp detection, the cells were detached with 0.25% trypsin-EDTA. A total of 8 × 10^3^ cells were plated on coverslips and incubated in 24-well plates (final volume: 500 µL) with tetracycline. After 24–72 h, the assay was performed. The extracellular recording medium contained 140 mM TEA–Cl, 2 mM MgCl_2_–6H_2_O, 10 mM CaCl_2_–2H_2_O, 10 mM HEPES and 5 mM D-glucose at pH 7.4, together with TEA-OH. The intracellular medium contained 120 mM CsCl, 1 mM MgCl_2_–6H_2_O, 10 mM HEPES, 10 mM EGTA, 0.3 mM Na_2_-GTP and 4 mM Mg-ATP at pH = 7.2, together with CsOH. The voltage stimulation protocol for the membrane clamp recording of Ca_v_2.2 calcium currents was as follows: When a whole-cell seal was formed, the cell membrane voltage was clamped at −80 mV. The clamp voltage was depolarized from −80 mV to +10 mV and maintained for 0.3 s, and data acquisition was repeated at 20 s intervals to detect the effect of the drug on the Ca_v_2.2 currents. Test data were acquired with the EPC 10 amplifier (HEKA) and saved in the PatchMaster software (HEKA, v2.15).

### 4.10. Animals

ICR mice (50%/50% male and female) weighing 18–22 g were purchased from Huafukang Beijing Bioscience Co., Ltd. (Beijing, China). The mice were provided with an appropriate growth environment with adequate water and food. The mouse experiments were carried out according to the ARRIVE guidelines and were approved by the State Key Laboratory of NBC Protection for Civilian (LAE-2022-08-001). 

### 4.11. ICV Injection Procedures

The mice were anesthetized with 5% isoflurane. The skulls of the mice were exposed, and the coordinates of the positions (0.46 mm posterior to bregma and 1 mm lateral to the midline) were determined using a brain stereotaxic (RWD Life Science Inc, Shenzhen, China). The position coordinates were pierced with a needle, and a brain catheter of 2.5 mm depth was implanted into the skull. The skull was fixed with surface screws, resin powder and adhesive. After surgery, the mice were housed separately in a single cage, and experiments were performed one week later. For the ICV injections, an inner tube attached to a polyethylene tube was inserted into the brain catheter of the mice, and the peptide drug or vehicle (1 µL) was injected at a rate of 0.5 µL/min and allowed to stand for 1 min after administration.

### 4.12. Acetic-Acid-Induced Writhing Test 

The mice were randomly grouped (50%/50% males and females). To compare the in vivo analgesic activities of MVIIA and Myr-MVIIA, the mice were first given equal doses (1 nmol/kg) of MVIIA and Myr-MVIIA in the lateral ventricles and saline as a control. Then, 30 min later, the mice were injected intraperitoneally with 1% acetic acid. The frequency of writhing was counted within 0–20 min after acetic acid injection. The writhing of the mice was characterized by abdominal muscle contraction, hind limb extension and body elongation.

The duration of analgesic activity of the peptides was tested as follows. The mice were injected with MVIIA at a dose of 1 nmol/kg (an excessively high a dose can cause tremors in the mice, seriously interfering with the results of the experiment) and Myr-MVIIA at a dose of 16 nmol/kg (Myr-MVIIA was used at a dose appropriate for self-assembly). Saline was injected into the control group. Each group was injected with either peptides or saline for different time intervals (1, 2, 4, 6, 8, 10 h) before acetic acid was administered intraperitoneally to the mice. The inhibition rate of writhing was expressed by the formula inhibition (%) = (Ns − Np) × 100/Ns, where Ns refers to the number of writhing movements in the mice after saline injection and Np refers to the number of writhing movements in the mice after peptide injection.

### 4.13. Coordinated Locomotion Test 

The mice were randomly grouped (50%/50% males and females). The rotarod test was used to evaluate the impacts of MVIIA (1 nmol/kg) and Myr-MVIIA (16 nmol/kg) on coordinated locomotion in the mice. The mice were trained for three consecutive days prior to the test by placing them on a rotarod at 10 rpm for 3 min. The mice were first injected (ICV) with MVIIA/Myr-MVIIA, and saline was injected as a control (6 mice per group). The rotarod mode was set to the uniform acceleration mode in advance so that the rotarod rotated from 5 rpm to 30 rpm with a uniform acceleration for 3 min. After 30, 60, 120 and 240 min from injection, the mice were perched on the rotarod (at 5 rpm) for 68 s. Then, the set instrument mode was adjusted, and the timing started. The time during which the mice stayed on the rotarod was recorded (maximum stay time was 3 min).

### 4.14. Tremor Test 

The mice were randomly grouped (50%/50% males and females). The mice were administered MVIIA and Myr-MVIIA peptides (1 nmol/kg and 16 nmol/kg, respectively) to evaluate the tremor intensity. The peptides were administered by ICV to the mice (*n* = 8). The tremor intensity was assessed at 30, 60, 120, 240 and 360 min after injection.

### 4.15. Peptide Stability in Human Sera

The stability of the peptides was tested using a method modified from a previously reported procedure [33]. Briefly, diluted human serum (10%) was centrifuged at 13,000× *g* for 15 min to separate the lipid layer from the serum, and the supernatant was incubated at 37 °C for 15 min. The initial peptide solution (10 mM) was diluted with the supernatant to a final concentration of 100 µM and incubated at 37 °C. At various time points (0–24 h), a 60 µL aliquot was removed and mixed with 60 µL of 80% aqueous acetonitrile at 4 °C for 10 min to terminate enzyme degradation. The final mixture was centrifuged to remove the precipitate, and the supernatant was analyzed by HPLC. The peak area of the sample at 0 min of reaction was set to a 100% peptide content, and the percentage of peptide was obtained according to the following formula: intact peptide (%) = integrated peak area at a fixed time point/integrated peak area of peptide at 0 min × 100. 

### 4.16. Statistical Analysis

Quantification and statistical analyses were carried out using Microsoft Office Excel and GraphPad Prism v8. The coordinated locomotion test results were analyzed using one-way ANOVA with Dunnett’s T3 multiple comparison test. The serum stability test results of the peptides were analyzed using *t*-tests. All data are presented as the mean ± standard error (SEM). Differences with *p*-values less than 0.05 were considered statistically significant.

## Data Availability

The data presented in this study are available on request from the corresponding author.

## Supplementary Materials

The following are available online at https://www.mdpi.com/article/10.3390/md21040229/s1, Table S1. Particle sizes of the peptides MVIIA and Myr-MVIIA; Figure S1: (A) HPLC of MVIIA (B) The molecular mass of the MVIIA was determined by ESI-MS; Figure S2: (A) HPLC of Myr-MVIIA (B) The molecular mass of the Myr-MVIIA was determined by ESI-MS.
