# Supplementary material for: Self-Assembly Nanostructure of Myristoylated ω-Conotoxin MVIIA Increases the Duration of Efficacy and Reduces Side Effects
Source: Mar Drugs. 2023 Apr 1;21(4):229. doi: 10.3390/md21040229 (PMC10144222; doi:10.3390/md21040229)
Supplement: Supplementary file 1 [file marinedrugs-21-00229-s001.zip › marinedrugs-2309971-supplementary-4.1.pdf]

Self-Assembly Nanostructure of Myristoylated ω-Conotoxin MVIIA Increases the Duration of Efficacy and Reduces Side Effects

Xiufang Ding [1], Yue Wang [1,2], Sida Zhang [1], Ruihua Zhang [1], Dong Chen [1], Long Chen [2], Yu Zhang [1], Shi-Zhong Luo [2], Jianfu Xu [1,*] and Chengxin Pei [1,*]

1 State key Laboratory of NBC Protection for Civilian, Beijing 102205, China

2 Beijing Key Laboratory of Bioprocess, College of Life Science and Technology, Beijing University of Chemical Technology, Beijing 100029, China

* Correspondence: jianfuxu2000@hotmail.com (J.X.); peichengxin@sklnbcpc.cn (C.P.)

Table S1. Particle sizes of the peptides MVIIA and Myr-MVIIA.

peptide	Particle Size (nm)	PDI
MVIIA	48.74 ± 16.07	0.514
Myr-MVIIA	301.83 + 98.09	0.400

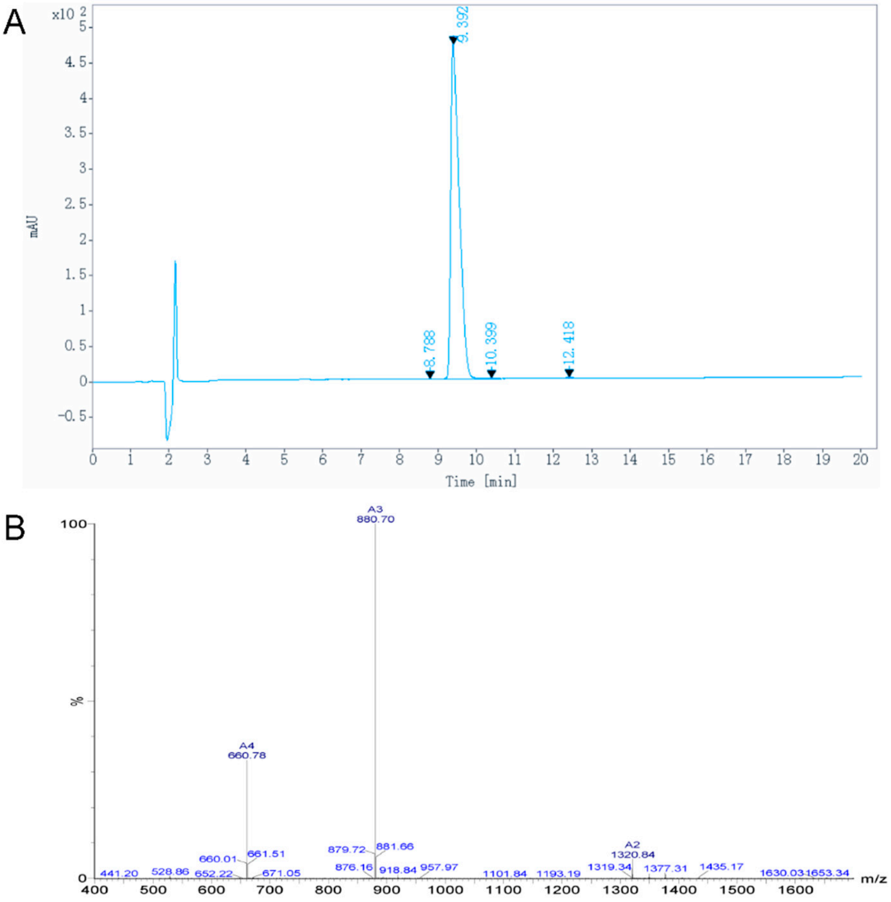

Figure S1. (A) HPLC of MVIIA (B) The molecular mass of the MVIIA was determined by ESI-MS. expected mass: 2639.2, observed mass: [M+2H]/2: 1320.84, [M+3H]/3: 880.70, [M+4H]/4: 660.78.

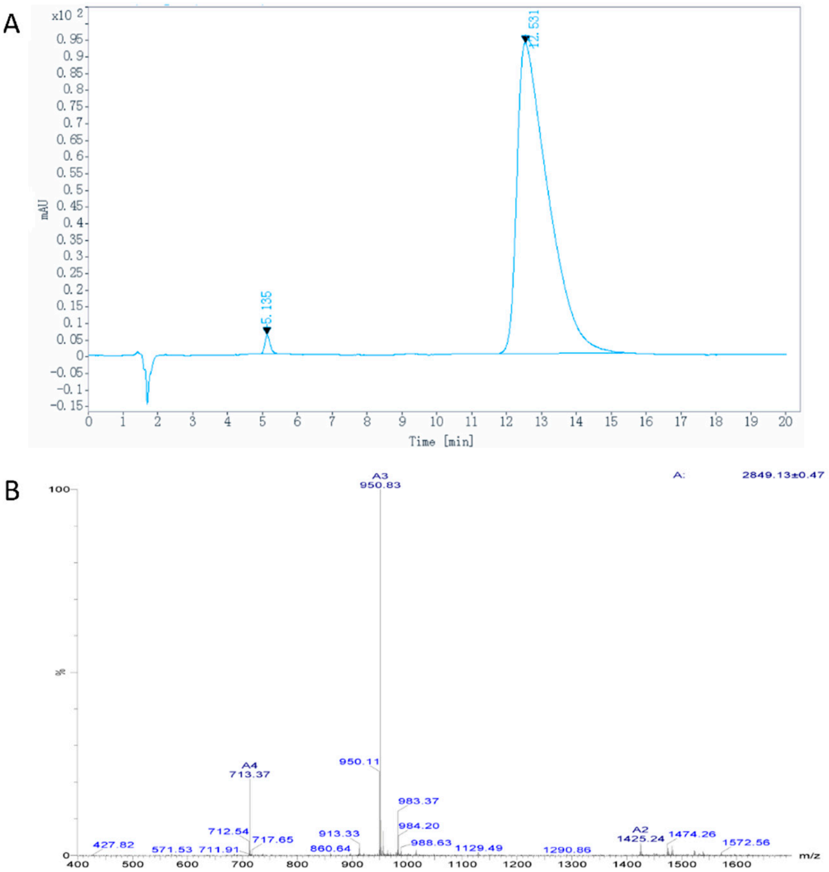

Figure S2. (A) HPLC of Myr-MVIIA (B) The molecular mass of the Myr-MVIIA was determined by ESI-MS. expected mass: 2849.5, observed mass: [M+2H]/2: 1425.24, [M+3H]/3: 950.83, [M+4H]/4: 713.37.
